# Infection during the first year in patients treated with CD19 CAR T cells for diffuse large B cell lymphoma

**DOI:** 10.1038/s41408-020-00346-7

**Published:** 2020-08-05

**Authors:** Kitsada Wudhikarn, M. Lia Palomba, Martina Pennisi, Marta Garcia-Recio, Jessica R. Flynn, Sean M. Devlin, Aishat Afuye, Mari Lynne Silverberg, Molly A. Maloy, Gunjan L. Shah, Michael Scordo, Parastoo B. Dahi, Craig S. Sauter, Connie L. Batlevi, Bianca D. Santomasso, Elena Mead, Susan K. Seo, Miguel-Angel Perales

**Affiliations:** 1 Adult Bone Marrow Transplant Service, Department of Medicine, Memorial Sloan Kettering Cancer Center, New York, NY USA; 2 Division of Hematology and Research Unit in Translational Hematology, Department of Medicine, Chulalongkorn University, Bangkok, Thailand; 3 Lymphoma Service, Department of Medicine, Memorial Sloan Kettering Cancer Center, New York, NY USA; 4 Department of Medicine, Weill Cornell Medical College, New York, NY USA; 5 Division of Hematology, Oncology and Hemato-Oncology Department, Fondazione IRCCS Istituto Nazionale dei Tumori and University of Milan, Milan, Italy; 6 Department of Biostatistics and Epidemiology, Memorial Sloan Kettering Cancer Center, New York, NY USA; 7 Department of Neurology, Memorial Sloan Kettering Cancer Center, New York, NY USA; 8 Critical Care Medicine Service, Department of Anesthesiology and Critical Care Medicine, Memorial Sloan Kettering Cancer Center, New York, NY USA; 9 Infectious Disease Service, Department of Medicine, Memorial Sloan Kettering Cancer Center, New York, NY USA

**Keywords:** B-cell lymphoma, Infectious diseases

## Abstract

CD19-targeted chimeric antigen receptor (CAR) T cell therapy is an effective treatment for diffuse large B cell lymphoma (DLBCL). In addition to cytokine release syndrome (CRS) and immune effector cell-associated neurotoxicity (ICANS), B cell aplasia and hypogammaglobulinemia are common toxicities predisposing these patients to infections. We analyzed 60 patients with DLBCL treated with FDA-approved CD19 CAR T cells and report the incidence, risk factors, and management of infections during the first year after treatment. A total of 101 infectious events were observed, including 25 mild, 51 moderate, 23 severe, 1 life-threatening, and 1 fatal infection. Bacteria were the most common causative pathogens. The cumulative incidence of overall, bacterial, severe bacterial, viral, and fungal infection at 1 year were 63.3%, 57.2%, 29.6%, 44.7%, and 4%, respectively. In multivariate analyses, the use of systemic corticosteroids for the management of CRS or ICANS was associated with an increased risk of infections and prolonged admission. Impaired performance status and history of infections within 30 days before CAR T cell therapy was a risk factor for severe bacterial infection. In conclusion, infections were common within the first 60 days after CAR T cell therapy, however, they were not associated with an increased risk of death.

## Introduction

CD19-directed chimeric antigen receptor (CAR) T cell is a major breakthrough that has revolutionized the treatment paradigm of relapsed/refractory (RR) diffuse large B cell lymphoma (DLBCL) over the past recent years^1,2^. Despite the significant anti-lymphoma activity, CD19 CAR T cells possess unique toxicities. Besides immune-mediated toxicities, B cell aplasia, and resultant hypogammaglobulinemia are common consequences of CD19 CAR T cell therapy, which put patients at risk for infectious complications^3,4^. Although there have been some initial reports on the infectious complications of CAR T cell therapy, most studies included patients treated in clinical trials or with multiple underlying B cell malignancies^5–10^. Currently, there are limited real-world data on infectious risks in patients treated with CD19 CAR T cell therapy for DLBCL. Moreover, little is known about proper prophylaxis and management strategies for these patients. Herein, we describe the pattern, incidence, impact of infections, including infection prophylactic strategies, in patients with DLBCL who received FDA-approved CAR T cell therapy at Memorial Sloan Kettering Cancer Center (MSKCC).

## Sample and methods

The study cohort included 60 consecutive patients with RR DLBCL who received FDA-approved CAR T cell therapy (axicabtagene ciloleucel—Yescarta; Kite Pharma, Santa Monica, CA or tisagenlecleucel—Kymriah; Novartis, Basel, Switzerland) at MSKCC between January 2018 and June 2019. Baseline clinical characteristics, patterns of antimicrobial prophylaxis, treatment of infection, and laboratory data, including blood count, CD4 lymphocyte, and immunoglobulin (Ig) level before lymphodepletion (LD) chemotherapy were abstracted from the electronic health records (EHR). Systemic bridging therapy were classified to intensive or non-intensive regimens. Intensive regimens included multi-agent immunochemotherapy e.g. CHOP-like, bendamustine-based, gemcitabine-based, high dose cytarabine-based, and ICE regimens. Non-intensive regimens included single-agent rituximab, immunomodulatory agent or small molecule inhibitor. The LD chemotherapy before CAR T cell infusion was selected based on recommended regimens in the package insert of each approved CAR T product. In patients who received axicabtagene ciloleucel, LD chemotherapy consisted of fludarabine 30 mg/m^2^, and cyclophosphamide 500 mg/m^2^ daily for 3 days. For patients receiving tisagenlecleucel, fludarabine 25 mg/m^2^ and cyclophosphamide 250 mg/m^2^ daily for 3 days or bendamustine 90 mg/m^2^ daily for 2 days was given for LD.

Prior to March 2019, there was no formal institutional guideline for antimicrobial prophylaxis or infection surveillance in patients treated with CAR T cell therapy, and treatment regimen was based on the autologous stem cell transplant (SCT) protocol or left to the primary physician’s preference. Standardized guidelines for CAR T cell patients were implemented in March 2019 and included acyclovir for herpes simplex virus (HSV) prophylaxis, fluconazole for antifungal prophylaxis, and trimethoprim/sulfamethoxazole or aerosolized pentamidine for *Pneumocystis jiroveci* prophylaxis (Table 1).

**Table 1:** Infection prophylaxis guideline for chimeric antigen receptor T cell patients at Memorial Sloan Kettering Cancer Center.

	Recommended agent	Duration
Antiviral prophylaxis	Acyclovir^a^ 400 mg orally twice daily	Commence with chemotherapy and continue for at least 6 months post-CAR T infusion
Anti-*Pneumocystis* prophylaxis^b^	Trimethoprim/Sulfamethoxazole 1 double-strengh tablet orally three times a week OR, if allergic or intolerant, Aerosolized pentamidine 300 mg monthly	Commence with chemotherapy and continue for 3 months post-CAR T infusion Consider extending duration beyond 3 months with persistent lymphopenia (CD4 < 200 cells/µL)
Antifungal prophylaxis	Fluconazole^a^ 200 mg orally daily	Commence with chemotherapy and continue until neutrophil recovery (ANC > 500 cells/µL for at least 3 days)
Antifungal prophylaxis for patients at high risk for mold infection (e.g., prednisone >20 mg for >2 weeks or equivalent)	Voriconazole^a,c^ 200 mg orally twice daily	

*CAR* chimeric antigen receptor, *ANC* absolute neutrophil count.

^a^Prophylaxis was converted to an intravenous formulation if patient was unable to tolerate oral intake.

^b^For patients unable to take sulfa or pentamidine, dapsone 100 mg daily or atovaquone 1500 mg daily were alternatives.

^c^Voriconazole was switched to micafungin 100 mg daily 48 h prior to and restarted 48 h after cyclophosphamide conditioning.

All infections were documented from the day of CAR T cell infusion through 1-year post-CAR T cell therapy, last follow-up, or relapse/progression, whichever came first. Infection events included both confirmed infections in which causative pathogens were identified, and probable infections diagnosed by the presence of fever plus localized physical exam and/or radiological findings. Any episode of culture-negative neutropenic fever in the absence of localized infection within the first 30 days after CAR T cell infusion was excluded from the analysis owing to the high probability of overlap with cytokine release syndromes (CRS). We classified types of infection according to causal pathogens, including bacterial, viral, fungal, and protozoal infection. Bacterial infections were further classified into organ-specific infection or bacteremia without localizing organ involvement. Infection severity was graded as mild, moderate, severe, life-threatening, or fatal, according to published criteria^5,11^. Mild infection was defined as not requiring antimicrobial therapy. Moderate infection entailed therapy with an oral antimicrobial medication. Severe infection was defined as infection requiring receipt of intravenous antimicrobial treatment. Life-threatening infection was defined as the presence of end-organ or cardiovascular compromise. Cumulative incidence of any infection, bacterial infection, severe bacterial infection, and viral infection were reported and separated by time after CAR T cell infusion (0–30 days, 31–100 days, 101–180 days, and 181–365 days). The data cutoff for statistical analysis was December 31, 2019.

### Statistical analysis

We reported continuous variables using median and range. Categorical data were presented as a percentage. Overall survival was analyzed by Kaplan–Meier methodology and infections were treated as time-dependent covariates. Cumulative incidence of time to the first infection was evaluated with progression of disease, relapse, and death from non-infection causes as competing events. Factors associated with infection were identified by univariate analysis using cause-specific hazard ratios and 95% confidence intervals. CRS, immune effector cell-associated neurotoxicity syndrome (ICANS), corticosteroid, tocilizumab, and intravenous immunoglobulin (IVIG) were treated as time-dependent covariates. *P*-values less than 0.10 were considered for multivariate analysis using cause-specific hazard ratios. All statistical analyses were performed by R program version 3.6.0. The cmprsk package was used for the cumulative incidence of infection. The institutional review board and the ethic committee of MSKCC granted approval for conducting the study.

## Results

### Baseline clinical characteristics

Table 2 summarizes the baseline clinical characteristics of the 60 patients in this cohort. The median age at the time of CAR T treatment was 63 years (19.5–85.9 years). Thirty-five patients (58%) had de novo DLBCL. Patients had a median of 4^2–9^ prior lines of treatment before CAR T cells, and 16 (26.7%) underwent prior hematopoietic cell transplantation. Thirty-eight patients (63.3%) received bridging therapy before CAR T cells (4 radiation therapy, 33 immunochemotherapy and 1 combined modality). Forty-three patients (71.7%) were treated with axicabtagene ciloleucel, and 17 (28.3%) received tisagenlecleucel. The median length of hospital stay for CAR T cell admission was 17 days (0–72 days). Thirteen patients (21.7%) received CAR T cell therapy after the institutional antimicrobial prophylactic protocol was implemented.

**Table 2:** Baseline characteristics of large B cell lymphoma treated with CD19 chimeric antigen receptor T cells.

Baseline parameters	*N* = 60 (%)
Median Age at Chimeric Antigen Receptor T Cell (range)	63 (19.5–85.9) years
Gender (Male:Female)	42:18
Histopathological diagnosis	
De novo diffuse large B cell lymphoma	35 (58.3)
Transformed indolent lymphoma	25 (41.7)
Stage	
Stage 1–2	14 (23.3)
Stage 3–4	38 (63.3)
Not available	8 (13.3)
ECOG Performance status	
0–1	44 (73.3)
2	10 (16.7)
3	2 (3.3)
Not available	4 (6.7)
Median number of treatment lines prior to CAR T cells (range)	3 (2–9)
Response to prior treatments	
Primary refractory disease	10 (16.7)
Relapsed disease	50 (83.3)
Presence of bulky disease	
Yes	9 (15.0)
No	45 (75.0)
Not available	6 (10.0)
History of hematopoietic stem cell transplant prior to CAR T cells	
Allogeneic hematopoietic stem cell transplant^a^	12 (20.0)
Autologous hematopoietic stem cell transplant^a^	5 (8.3)
No	44 (73.3)
Bridging treatment before CAR T cell	
High-Intensity systemic therapy	23 (38.3)
Low-Intensity systemic therapy^b^	11 (18.3)
Radiotherapy^b^	5 (8.3)
No bridging therapy or systemic corticosteroid	22 (36.7)
History of Infection within 30 days before CAR T cell	24 (40.0)
Lymphodepletion chemotherapy	
Fludarabine cyclophosphamide	57 (95.0)
Bendamustine	3 (95.0)
CAR T cell product	
Axicabtagene ciloleucel	43 (71.7)
Tisagenlecleucel	17 (28.3)

E*COG* Eastern Cooperative Oncology Group, *CAR* chimeric antigen receptor.

^a^One patient had both autologous and allogeneic hematopoietic stem cell transplant.

^b^One patient received combined non-intensive systemic and radiation therapy.

### Baseline infection and antimicrobial prophylaxis

Nineteen patients (31.7%) received systemic antibacterial treatment for an infection within 30 days before the CAR T cell infusion. Three of these patients continued antibiotics through the admission of CAR T cell therapy. Thirty-one patients (51.7%) received antibacterial prophylaxis (Supplementary Table S1). All patients received antiviral prophylaxis for HSV, and 6 received entecavir due to the positive hepatitis B core antibody. Fifty-five patients (91.7%) were given prophylaxis for *Pneumocytis jiroveci*. Forty-eight patients (80%) received antifungal prophylaxis, 26 were initiated before CAR T cell infusion whereas the other 22 had antifungal prophylaxis started once absolute neutrophil count (ANC) was less than 500 µ/mL (median time from CAR T infusion to antifungal prophylaxis initiation of 7 days).

### Recovery of leukocytes and immunoglobulin levels (Table 3)

Baseline median ANC and absolute lymphocyte counts (ALC) before LD chemotherapy were 3850 (200–10,600) and 600 (100–2700) cells/µL, respectively. Two patients had grade 4 neutropenia (ANC < 500 cells/µL) before LD therapy, as defined by CTCAE version 5.0^12^. Forty-seven of the remaining 58 patients (81.0%) developed neutropenia grade 4 after LD therapy. The median duration of neutropenia grade 4 was 12 days (3–66 days). Ten patients had grade 4 neutropenia after day 30 (5 of which had persistent grade 4 neutropenia from the first 30 days). Thirty patients required at least one dose of growth factor support after CAR T cell therapy. Grade 3–5 lymphopenia beyond day 30 after CAR T cell was observed in 35 patients (58.3%). Of 19 patients who had lymphocyte subset analysis at day 30, all had B cell aplasia and the median CD4^+^ lymphocyte count was 116 cells/µL (41–630 cells/µL). Supplementary Table S2 summarizes the status of leukocyte subset reconstitution after CAR T cell infusion.

**Table 3:** Baseline cytokine and immune function of large B cell lymphoma treated with CD19 chimeric antigen receptor T cells.

	*N* (%)
Baseline absolute neutrophil count prior to lymphodepletion chemotherapy	
Less than 500 cells/µL	1 (1.7)
500–1000 cells/µL	1 (1.7)
1000 cells/µL or higher	58 (96.7)
Baseline absolute lymphocyte count prior to lymphodepletion chemotherapy	
Less than 500 cells/µL	20 (33.3)
500–1000 cells/µL	29 (48.3)
1000 cells/µL or higher	11 (18.3)
Baseline CD4^+^ lymphocyte count prior to lymphodepletion chemotherapy (in 19 patients)	
Less than 200 cells/µL	8 (42.1)
200–500 cells/µL	10 (52.6)
500 cells/µL or higher	1 (5.3)
Baseline immunoglobulin G level prior to lymphodepletion chemotherapy	
Less than 400 mg/dL	15 (25.0)
400 mg/dL or higher	45 (75.0)
Baseline lactate dehydrogenase level prior to lymphodepletion chemotherapy	
Normal	30 (50.0)
Elevate x1 to x3 upper normal limit	24 (40.0)
Higher than x3 upper normal limit	6 (10.0)
Median baseline interleukin-6 level prior to CAR T cell infusion (range, pg/mL)	11.8 (2.5–246.0)
Median baseline ferritin level prior to CAR T cell infusion (range, ng/mL)	302 (8–8201)
Median baseline C-reactive protein level prior to CAR T cell infusion (range, mg/L)	1.6 (0.1–27.5)
Median baseline procalcitonin level prior to CAR T cell infusion (ng/mL)	0.1 (0–3.5)

*CAR* chimeric antigen receptor.

**Fig. 1:**
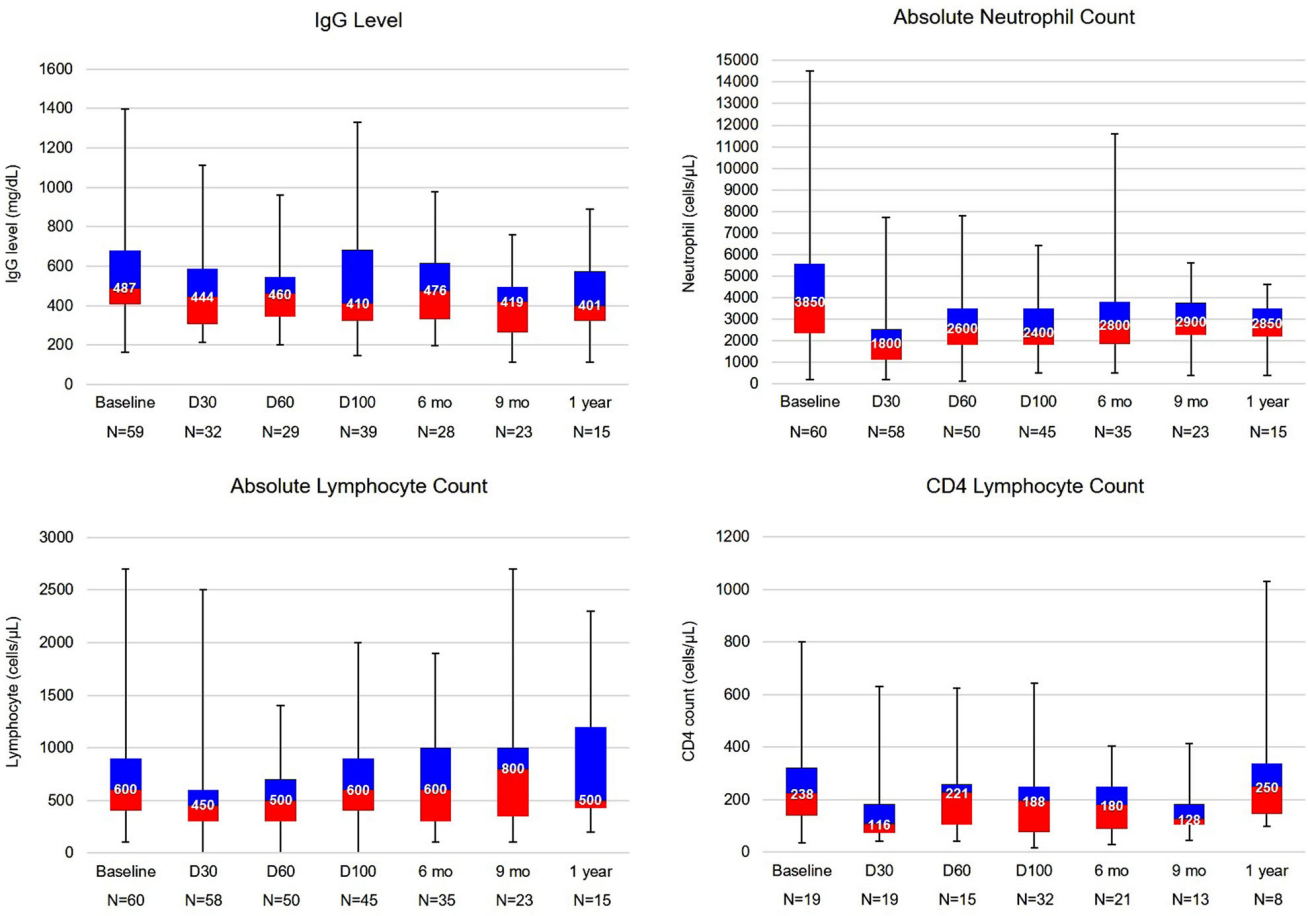
Baseline immune function and immune recovery after CAR T cell therapy. Immune status at baseline before lymphodepletion and recovery by time post chimeric antigen receptor T cell therapy (number in the boxplot indicates median value of each parameter at each timepoint). **a** Immunoglobulin G (IgG) Level. **b** Absolute neutrophil count. **c** Absolute lymphocyte count. **d** CD4 lymphocyte count. D Day, mo Month.

In 59 patients with available baseline IgG level before LD chemotherapy, the median IgG level was 487 mg/dL (163–1399 mg/dL), and 15 patients (25%) had baseline hypogammaglobulinemia (IgG ≤ 400 mg/dL). Immunoglobulin levels were checked at day 30 after CAR T cell therapy in 32 patients; 14 (43.8%) had hypogammaglobulinemia. An additional 12 patients (37.5%) developed hypogammaglobulinemia at later follow-up timepoints. Nineteen patients (31.7%) received at least 1 dose of IVIG replacement, including 10 (52.6%) who had IVIG after history of recurrent infections. Figure 1 illustrates patterns of leukocyte and IgG level during the CD19 CAR T cell treatment course.

### CRS and ICANS

CRS was observed in 48 patients (80%) (grade ≥ 3 in 7 patients; 11.7%) at a median onset of 2 days after CAR T cell infusion (0–11 days). ICANS was observed in 24 patients (40%) and was grade ≥3 in 13 patients. The median onset of ICANS was 5 days after infusion. Of patients who developed CRS or ICANS, 25 (53.2%) received systemic corticosteroid with a median duration of 4 days (1–58 days). The median prednisone equivalent dose intensity of corticosteroid was 1.12 mg/kg/day (0.33–4.23 mg/kg/day) with the corresponding median cumulative dose of 380 mg (66.7–4586 mg) or 8.1 mg/kg (0.33–69.6 mg/kg) prednisone equivalent. Patients who received systemic corticosteroid had longer median hospital stay than patients who did not (27 days vs. 14 days, *P* < 0.001).

Baseline c-reactive protein, procalcitonin, and IL-6 before CAR T cell infusion were elevated in 52 (86.7%), 4 (7.4%), and 38 (63.3%) patients, respectively. The median peak procalcitonin level was 0.46 (0.05–27.65) µg/L. The trends of procalcitonin and IL-6 are shown in Supplementary Figs. S1 and S2, respectively.

### Incidence, characteristics, and patterns of infection after CAR T cell therapy

Fifty-two of 60 patients (86.7%) developed neutropenic fever within the first 30 days after CAR T cell infusion. With the median follow-up of 6 months (0.8–12 months), after excluding neutropenic fever without identified pathogen or localizing organ, there was a total of 101 infection events (60 bacterial, 38 viral, 2 fungal, and 1 protozoal) in 40 patients during the entire study period. Pathogenic organism were identified in 73 infection events (72%) (60% of bacterial, 92.1% of viral, 50% of fungal, and 100% of protozoal infection). Thirty-seven infection episodes (34.6%) occurred within the first 30 days after CAR T cell therapy. Of 101 events, 23 (22.8)% infections were classified as severe, 1 (1.0%) as life-threatening (*Escherichia coli* biliary sepsis), and 1 (1.0%) fatal (influenza A pneumonia). Of all 101 infection events, 32 occurred during the initial CAR T cell admission. Among the other 69 infection episodes (bacterial; *n* = 42, viral; *n* = 26, fungal; *n* = 1), which occurred following hospital discharge from CAR T cell therapy, 21 (bacterial; *n* = 14, viral; *n* = 6, fungal; *n* = 1) required hospital readmission with the median hospital stay of 5 (2–37) days.

**Fig. 2:**
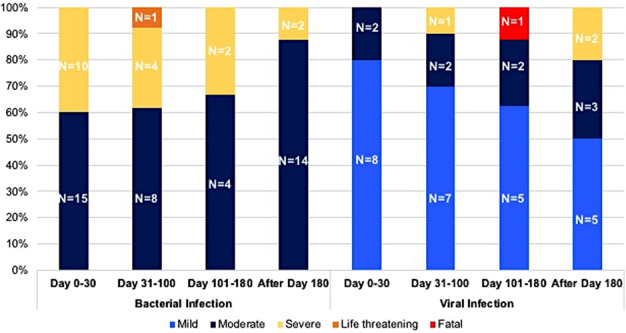
Infections after CD19 CAR T cell therapy. Distribution of bacterial and viral infection by time post chimeric antigen receptor T cell and severity.

**Fig. 3:**
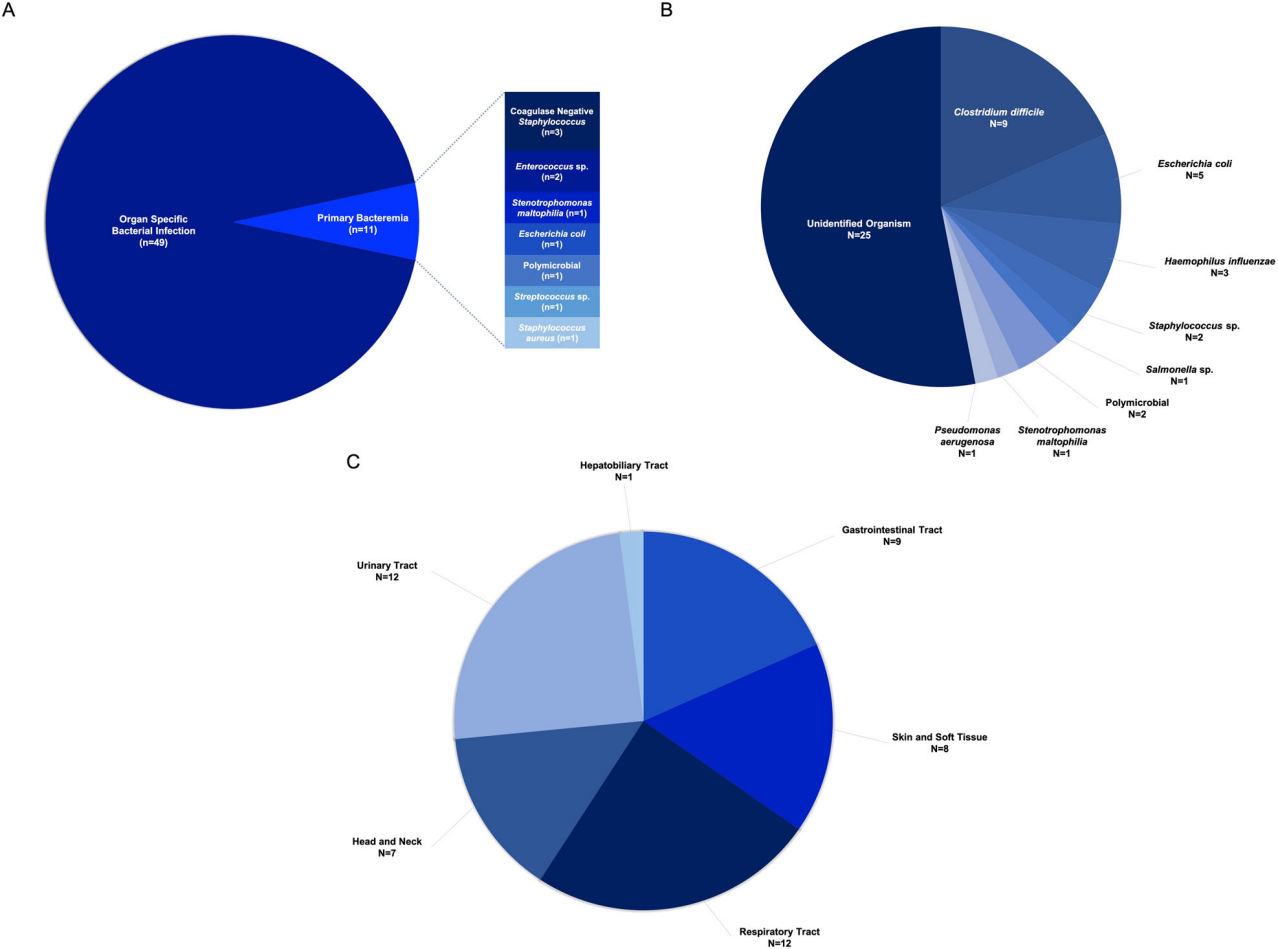
Distribution of bacterial infection. **a** By localization—primary bacteremia vs. Localized infection. **b** Identified organism in localized bacterial infection. **c** Localized bacterial infection by involved organs.

**Fig. 4:**
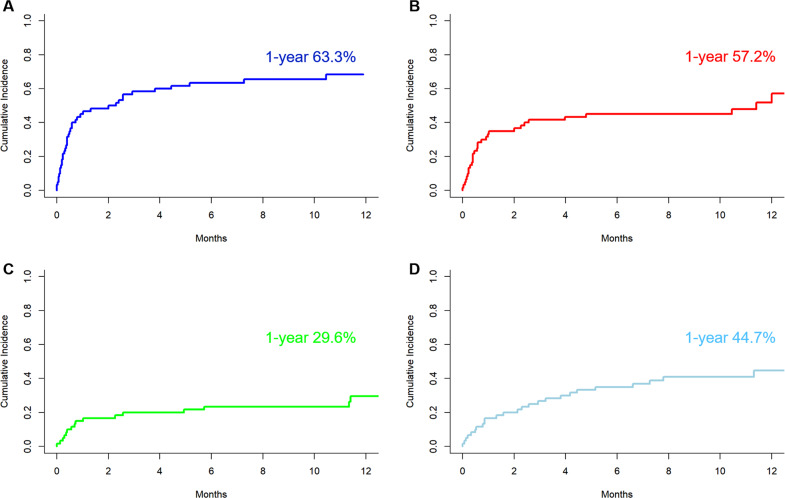
Cumulative Incidence of infection. **a** Any infection. **b** Bacterial Infection. **c** Severe Bacterial Infection. **d** Viral Infection.

Figure 2 shows the distribution of bacterial or viral infection at each period post-CAR T cell infusion. The distribution of organ involvement and causative organism of bacterial infection are shown in Fig. 3 and Supplementary Table S3. The 1-year cumulative incidence of all infections, bacterial, viral, and fungal infections were 63.3, 57.2, 44.7%, and 4.0%, respectively (Fig. 4, Table 4).

**Table 4:** Cumulative incidence of infection by causative pathogens at different follow up after chimeric antigen receptor T cell therapy.

	1 month (95% CI)	3 months (95% CI)	6 months (95% CI)	1 year (95% CI)
Overall Infection	45.0 (32.0–57.1)	58.3 (44.6–69.8)	61.7 (46.5–71.3)	63.3 (49.5–74.3)
Bacterial Infection	33.3 (21.7–45.4)	41.7 (29.0–53.8)	45.0 (32.0–57.1)	57.2 (39.3–71.6)
Severe Bacterial Infection	15.0 (7.30–25.2)	20.0 (11.0–31.0)	23.3 (13.5–34.7)	29.6 (17.4–42.9)
Viral Infection	16.7 (8.50–27.2)	26.7 (16.2–38.3)	35.0 (23.1–47.1)	44.7 (30.2–58.3)
Fungal Infection	1.7 (0.1–7.9)	1.7 (0.1–7.9)	1.7 (0.1–7.9)	4.0 (7.0–12.3)

*CI* confidence interval.

### Infection within the first 30 days of CAR T cell infusion

Of 37 infection episodes that occurred within the first 30 days after CAR T cell infusion (Supplementary Tables S3–S6), bacterial infections were the most frequent with 25 events (15 moderate, 10 severe) in 20 patients. The median onset of the first bacterial infection was day 12 (0–30). A total of 15 events were organ-specific infections, whereas 10 were primary bacteremias. Organisms were identified in 19 definite infectious episodes with *Clostridium difficile* (colitis) being the most common causative bacterial pathogen (*n* = 7). The other 6 events were probable infections (including 3 lobar pneumonia, 3 soft tissue infection). Piperacillin/tazobactam was the most common empirical anti-bacterial agent for neutropenic fever during the first 30 days in 38 patients (63.3%), followed by cefepime in 7 patients.

Ten viral infections occurred during the first 30 days (8 mild, 2 moderate) with the median onset at day 8 (0–26 days). Viral pathogens included respiratory syncytial virus (*n* = 5), cytomegalovirus (*n* = 2), polyoma BK virus (*n* = 2), and norovirus (*n* = 1). Both cytomegalovirus infection were viremia without end organ involvement. There was 1 probable invasive aspergillosis pulmonary infection (elevated serum galactomannan antigen and consistent radiographic imaging), and 1 protozoal infection (*Cryptosporidium parvum*).

### Infection after day 30 post CAR T cell infusion

Thirty-five bacterial infections were observed in 16 patients after day 30, including 12 events during day 31–100, 7 during day 101–180, and 16 beyond day 180 (Supplementary Tables S3–S6). Six of these 16 patients had previous bacterial infection within the first 30 days. Among 35 bacterial infections, there were 26 moderate, 8 severe, and 1 fatal infection. Ninety-seven percent were organ-specific infections with urinary tract infection being the most common presentation (*n* = 11). Of 28 viral infections, 10 occurred during day 31–100, 8 occurred during day 101–180, and 10 occurred after day 180. Approximately 50% of viral infections were of mild severity (*n* = 17). Respiratory tract infections (*n* = 21) were the most common with rhinovirus being the most frequently recovered. There were 1 cytomegalovirus reactivation (viremia without organ dysfunction), 2 BK virus cystitis, and 2 herpes zoster reactivation (both of whom were on acyclovir prophylaxis). *Pneumocystis jiroveci* infection was identified in 1 patient at 9 months after CAR T cell infusion (4 months after pentamidine prophylaxis discontinuation).

### Risk factors associated with infection

In the univariate analysis, impaired baseline performance status, ICANS grade ≥2, and systemic corticosteroid exposure after CAR T cell infusion were associated with a higher incidence of overall infection (Table 5). In multivariate analysis, systemic corticosteroid was the only risk factor of infectious complications. Impaired performance status and previous infection 30 days before LD chemotherapy was independent predictors for severe bacterial infection (HR 3.98, 95% CI 1.3–12.2). Patients with low IgG before LD chemotherapy had higher risk of viral infection after CAR T cells (HR 5.7, 95% CI 2.3–14.3; Supplementary Table S7), however, IVIG replacement did not decrease the incidence of infection. CRS, tocilizumab administration, and procalcitonin were not associated with infection or severe bacterial infection. The incidence of infection was comparable between 47 patients who received CAR T cell therapy before March 2019 and 13 patients who were treated with CAR T cells after March 2019, when the standardized antimicrobial prophylaxis guideline was implemented (HR 1.22, 95% CI 0.6–2.5).

**Table 5:** Cox proportional hazard regression analysis for factors associated with all infection and severe bacterial infection after chimeric antigen receptor T cell therapy.

	Univariate analysis		Multivariate analysis	
	Hazard ratio (95% confidence interval)	*P*-value	Hazard ratio (95% confidence interval)	*P*-value
All Infection				
Age (≥60 vs. <60 years)	1.06 (0.55–2.02)	0.90	N/A	N/A
CART product (Tisagenlecleucel vs. Axicabtagene ciloleucel)	0.70 (0.33–1.48)	0.40	N/A	N/A
Performance status (≥2 vs. 0–1)	2.15 (1.06–4.37)	0.03	1.87 (0.91–3.84)	0.09
Transplant prior to CAR T cell therapy (Yes vs. No)	0.57 (0.26–1.24)	0.20	N/A	N/A
Infection before CAR T cell therapy (Yes vs. No)	0.84 (0.44–1.59)	0.60	N/A	N/A
Baseline lactate dehydrogenase before lymphodepletion (normal vs. high)	1.13 (0.60–2.10)	0.70	N/A	N/A
Baseline immunoglobulin G (<400 vs. ≥400 mg/dL)	1.76 (0.86–3.58)	0.12	N/A	N/A
Cytokine release syndromes (grade ≥ 3 vs. grade 0–2)	0.86 (0.30–2.43)	0.77	N/A	N/A
Immune effector cell neurotoxicities (grade ≥ 2 vs. grade 0–1)	2.27 (1.10–4.71)	0.03	N/A	N/A
Systemic corticosteroid during CAR T cell (Yes vs. No)	2.18 (1.08–4.41)	0.03	2.22 (1.05–4.67)	0.03
Tocilizumab during CAR T cell (Yes vs. No)	1.20 (0.60–2.40)	0.61	N/A	N/A
Severe bacterial infection				
Age (≥60 vs. <60 years)	0.48 (0.18–1.28)	0.14	N/A	N/A
CART product (Tisagenlecleucel vs. Axicabtagene ciloleucel)	0.62 (0.18–2.18)	0.50	N/A	N/A
Performance status (≥2 vs. 0–1)	3.69 (1.34–10.2)	0.01	2.84 (1.0–8.06)	0.05
Transplant prior to CAR T cell therapy (Yes vs. No)	0.89 (0.29–2.75)	0.80	N/A	N/A
Infection before CAR T cell therapy (Yes vs. No)	4.69 (1.60–13.7)	0.005	3.98 (1.30–12.20)	0.01
Baseline lactate dehydrogenase before lymphodepletion (Normal vs. High)	0.78 (0.29–2.13)	0.60	N/A	N/A
Baseline Immunoglobulin G before lymphodepletion (<400 vs. ≥400 mg/dL)	1.86 (0.67–5.12)	0.20	N/A	N/A
Cytokine release syndromes (grade ≥ 3 vs. grade 0–2)	2.18 (0.62–7.73)	0.22	N/A	N/A
Immune effector cell neurotoxicities (grade ≥ 2 vs. grade 0–1)	2.47 (0.87–7.03)	0.09	N/A	N/A
Systemic corticosteroid during CAR T cell (Yes vs. No)	2.53 (0.89–7.20)	0.08	N/A	N/A
Tocilizumab during CAR T cell (Yes vs. No)	1.86 (0.66–5.26)	0.24	N/A	N/A

*CAR* chimeric antigen receptor, *N/A* not applicable.

### Impact of infection on patients’ survival outcomes

Of all infectious complications, one resulted in death attributed to influenza pneumonia despite a 10-day course of oseltamivir treatment. There was no association between infectious complications and mortality risk in CAR T cell-treated patients when analyzed by univariate cox regression.

## Discussion

Our study reported comprehensive real-world data on infectious complications in DLBCL patients treated with commercially available CD19 CAR T cell products. Data from the pivotal studies of CD19 CAR T cell therapy in DLBCL demonstrated an incidence of 15–30% for severe infection^13–15^. The incidence of overall infection in our study was comparable to these landmark trials. Moreover, the patterns of infection in our cohort were similar to the findings from previous reports^5,7,10^. Bacterial and viral infections were commonly observed, with bacteria being the most common pathogen, especially during the first 30 days^5,7,10^. Recently, Cordeiro and colleagues reported the incidence of adverse events beyond day 90 from CAR T cell infusion^16^ and described a relatively low incidence of late infections (2.08 per patient-year), with most being of mild to moderate severity. Our study observed similar results with 71% of all infections considered mild to moderate. Upper respiratory tract infections were the most common infectious events. Serious infections occurred in 23.4% of DLBCL patients treated with commercial CD19 CAR T cell products; nonetheless, most infections were manageable and infection-related mortality was low similar to the results of previous reports^5,7,17^. In our study, one patient died from influenza A pneumonia at day +159 despite treatment with oseltamivir. Fungal infection was uncommon in patients treated with CAR T cells likely due to short duration of neutropenia^5,18^. One patient had *Pneumocystis jirovecii* pneumonia 4 months after pentamidine prophylaxis was stopped. Retrospectively, the patient’s CD4 count was 44 cells/µL at the time of infection, thus emphasizing the importance of implementing immune monitoring protocols to guide the duration of antimicrobial prophylaxis in these patients.

General practice for infection prophylaxis in patients treated with CAR T cell are heterogeneous and vary by institutions^19^. The recent European guidelines for antimicrobial prophylaxis and IVIG replacement in patients treated with CAR T cells^20^ was primarily based on the evidence from SCT patients^21^. Currently, the appropriate prophylactic approach in these patients remains unknown and requires further understanding of the immune reconstitution pattern and longer follow-up data. B cell aplasia is a well-known “off tumor and on target” phenomenon after CD19 CAR T cell therapy contributing to hypogammaglobulinemia in these patients. A quarter of patients in our cohort had IgG < 400 mg/dL, and 60% had IgG < 600 mg/dL. This finding was comparable to data from the JULIET trial^14^, which highlighted the baseline humoral immune defect in these patients. However, there are data showing persistent long-lived plasma cells after CAR T cell therapy^22^. In addition, Hill and colleagues recently demonstrated preserved anti-viral humoral immune response in patients treated with CD19 CAR T cells^23^. The authors reported sustained anti-measle IgG level independent of the total IgG level in 95% of patients. Moreover, overall anti-virome was preserved in most patients. Moreover, there was a low incidence of viral infection after day 90. In our study, there were 18 viral infectious episodes in 15 patients after day 100 with mild respiratory tract infection as the most common presentation similar to previous reports. Interestingly, we observed an increased risk of viral infection in patients with hypogammaglobulinemia, but no such correlation was seen with other types of infection (supplementary data). We hypothesize that IgG deficiency at baseline might indicate pre-existing depleted plasma cell and antibody repertoire, which may have a more critical impact on the ability to mount viral-specific neutralizing IgG and predispose patients to infection after CAR T cell therapy. The significance of hypogammaglobulinemia on the risk of infection in patients treated with CD19 CAR T cells warrants further study. In the ELIANA trial, all pediatric patients with precursor B acute lymphoblastic leukemia (ALL) received IVIG replacement. In contrast, the proportion of IVIG replacement among DLBCL patients treated in pivotal studies was lower and ranged around 20–60%^14,15,24^. Only 30% of our patients received IVIG replacement, of which half had a history of preceding recurrent infection after CAR T cell therapy. The primary malignancy probably has a critical contribution to the risk of infection attributed to underlying immune function and kinetics of immune recovery. Data from patients with B-ALL indicated that CD8^+^ lymphocyte recovered early whereas CD4^+^ lymphocyte had delayed recovery after CAR T cell therapy^25^. Further studies on infection prophylaxis, immunization, and immune reconstitution in CAR T cell-treated patients are warranted.

In our study, we identified systemic corticosteroid as a predictor for infection after CAR T cell therapy whereas history of infection within 30 days before CAR T cell infusion was associated with severe bacterial infection, which may contribute to longer hospital stays in these patients. This observation is similar to another previous retrospective study^8^. We did not see an association between CRS and infectious complications in our lymphoma cohort. Park and colleagues previously demonstrated severe CRS (grade ≥ 3) as a risk factor for bacterial infection in adult B-ALL treated with CD19 CAR T cells^7,10^. Other risk factors for infection after CAR T cell therapy shown by previous studies included higher number of prior treatments, higher doses of CAR T cells, older age, previous history of infection, and CD22-specific CAR T cells^5,8^. Finally, a recent study described the association between double peak IL-6 pattern (second surge of serum IL-6 after initial normalization) and life-threatening infection^26^. Along with the current interest of anti-IL6 therapy in severe acute respiratory syndrome coronavirus 2 patients with respiratory failure^27–34^, it is at least worth noting that we did not observe an association between the use of tocilizumab and infections in this cohort.

Our study has unique strengths. We comprehensively analyzed the real-world data on patterns of infection, detailed information on relevant immune status and prophylactic strategies during the first year after CAR T cell therapy in patients with DLBCL from a dedicated lymphoma patient cohort. We acknowledge several limitations of this study. Besides its retrospective nature, immune function monitoring and infection prophylaxis were not prospectively studied in a systematic manner. Lastly, the relatively small number of patients included in the study could limit its statistical power.

In summary, infection is common in DLBCL patients treated with CD19 CAR T cells. However, most events occurred early after CAR T cell therapy and were largely manageable. The mechanism of infection in these patients is complex and multifactorial. Appropriate infection prophylaxis in these patients remain to be determined, and prospective clinical trials are warranted.

## Supplementary information

Supplementary Tables
