# Supplementary material for: Infection during the first year in patients treated with CD19 CAR T cells for diffuse large B cell lymphoma
Source: Blood Cancer J. 2020 Aug 5;10(8):79. doi: 10.1038/s41408-020-00346-7 (PMC7405315; doi:10.1038/s41408-020-00346-7)

**Supplementary Table S1:** Detail of antimicrobial prophylaxis and immunoglobulin replacement

|  | **N=60 (%)** |
| --- | --- |
| **Antibiotic prophylaxis**   - Fluoroquinolone - Beta-lactam - No prophylaxis | 27 (45.0)  4 (6.7)  29 (48.3) |
| **Antiviral prophylaxis**   - Herpes simplex virus prophylaxis (Acyclovir) - Hepatitis B virus prophylaxis (Entecavir)* | 60/60 (100.0)  6/6 (100.0) |
| **Antifungal prophylaxis**   - Azole - Micafungin - No prophylaxis | 42 (70.0)  6 (10.0)  12 (20.0) |
| ***Pneumocystis jiroveci* prophylaxis**   - Trimethoprim/Sulfamethoxazole - Pentamidine - Atovaquone - No prophylaxis | 13 (21.7)  40 (66.7)  2 (3.3)  5 (8.3) |
| **Intravenous immunoglobulin replacement**   - Primary prophylaxis - Secondary prophylaxis - No prophylaxis | 9 (15.0)  10 (16.7)  41 (68.3) |

* 6 patients received hepatitis B virus re-activation prophylaxis

**Supplementary Table S2:** Leukocyte subset recovery at different timepoints

|  | **Absolute neutrophil count (cells/µL)** | | | | | **Absolute lymphocyte count (cells/µL)** | | | | | | | | |
| --- | --- | --- | --- | --- | --- | --- | --- | --- | --- | --- | --- | --- | --- | --- |
|  | **Baseline**  **N=60** | **Day 30**  **N=58** | **Day 100**  **N=45** | **Month 6**  **N=35** | **Year 1**  **N=15** | **Baseline**  **N=60** | **Day 30**  **N=58** | | **Day 100**  **N=45** | | **Month 6**  **N=35** | | **Year 1**  **N=15** | |
| **Median** | 3850 | 1800 | 2400 | 2800 | 2850 | 600 | 450 | | 600 | | 600 | | 500 | |
| **Min** | 200 | 0 | 500 | 500 | 400 | 100 | 0 | | 0 | | 100 | | 200 | |
| **Max** | 14500 | 7700 | 6400 | 11600 | 4600 | 2700 | 2500 | | 2000 | | 1900 | | 2300 | |
| **N with count > 500/µL (%)** | 58  (96.7%) | 48  (82.8%) | 43  (95.6%) | 34  (97.1%) | 14  (93.3%) | 31  (51.7%) | 21  (36.2%) | | 27  (60.0%) | | 20  (57.1%) | | 6  (40.0%) | |
|  | **CD4+ T lymphocyte (cells/µL)** | | | | | **CD19+ B lymphocyte (cells/µL)** | | | | | | | | |
|  | **Baseline**  **N=19** | **Day 30**  **N=19** | **Day 100**  **N=32** | **Month 6**  **N=21** | **Year 1**  **N=8** | **Baseline**  **N=19** | | **Day 30**  **N=19** | | **Day 100**  **N=32** | | **Month 6**  **N=21** | | **Year 1**  **N=8** |
| **Median** | 238 | 116 | 189 | 180 | 251 | 0 | | 0 | | 0 | | 0 | | 0 |
| **Min** | 36 | 41 | 16 | 30 | 99 | 0 | | 0 | | 0 | | 0 | | 0 |
| **Max** | 800 | 630 | 643 | 403 | 1030 | 270 | | 0 | | 253 | | 342 | | 0 |
| **N with count > 200/µL (%)** | 11  (57.9%) | 3  (15.8%) | 15  (46.9%) | 10  (28.6%) | 5  (62.5%) | 1  (5.3%) | | 0  (0.0%) | | 1  (3.1%) | | 1  (4.8%) | | 0  (0.0%) |
| **N with B cell aplasia (%)** | N/A | N/A | N/A | N/A | N/A | 12 | | 19 | | 29 | | 18 | | 0 |

N/A: Not Applicable

**Supplementary Table S3:** Localized Bacterial Infection as stratified by organ and causative organisms

| **Duration** | **Localization** | **Organism** |
| --- | --- | --- |
| **First 30 days** | Gastrointestinal tract (n=8)  Skin and soft tissue (n=4)  Respiratory tract (n=2)  Head and neck (n=1) | *Clostridium difficile* (n=7)  *Salmonella* sp. (n=1)  Polymicrobial (n=1  Unidentified organism (n=6) |
| **After day 30** | Urinary tract (n=12)  Respiratory tract (n=10)  Head and neck (n=6)  Skin and soft tissue (n=4)  Gastrointestinal tract (n=1)  Hepatobiliary tract (n=1) | *Escherichia coli* (n=5)  *Haemophilus influenzae* (n=3)  *Clostridium difficile* (n=2)  *Staphylococcus* sp. (n=2)  *Pseudomonas aeruginosa* (n=1)  Polymicrobial (n=1)  *Stenotrophomonas maltophilia* (n=1)  Unidentified organism (n=19) |

**Supplementary Table S4:** Distribution of infection by severity at different timepoints after CAR T cell therapy

|  | **Day 0 – Day 30** | **Day 31 – Day 100** | **Day 101 – Day 180** | **After Day 180** |
| --- | --- | --- | --- | --- |
| **All infection (n=101)** | 37 (36.6%) | 23 (22.8%) | 14 (13.9%) | 27 (26.7%) |
| **Bacterial Infection (n=60)**   - Mild - Moderate - Severe - Life threatening - Fatal | 0 (0)  15 (25.0%)  10 (16.7%)  0 (0)  0 (0) | 0 (0)  8 (13.3%)  4 (6.7%)  1 (1.7%)  0 (0) | 0 (0)  4 (3.7%)  2 (3.3%)  0 (0)  0 (0) | 0 (0)  14 (23.3%)  2 (3.3%)  0 (0)  0 (0) |
| **Viral Infection (n=38)**   - Mild - Moderate - Severe - Life threatening - Fatal | 8 (21.0%)  2 (5.3%)  0 (0)  0 (0)  0 (0) | 7 (18.4%)  2 (5.3%)  1 (2.6%)  0 (0)  0 (0) | 5 (13.2%)  2 (5.3%)  0 (0)  0 (0)  1 (2.6%) | 5 (13.2%)  3 (7.9%)  2 (5.3%)  0 (0)  0 (0) |
| **Fungal Infection (n=2)**   - Mild - Moderate - Severe - Life threatening - Fatal | 0 (0)  0 (0)  1 (50.0%)  0 (0)  0 (0) | 0 (0)  0 (0)  0 (0)  0 (0)  0 (0) | 0 (0)  0 (0)  0 (0)  0 (0)  0 (0) | 0 (0)  0 (0)  1 (50.0%)  0 (0)  0 (0) |
| **Other Infection (n=1)**   - Mild - Moderate - Severe - Life threatening - Fatal | 0 (0)  1 (100.0%)  0 (0)  0 (0)  0 (0) | 0 (0)  0 (0)  0 (0)  0 (0)  0 (0) | 0 (0)  0 (0)  0 (0)  0 (0)  0 (0) | 0 (0)  0 (0)  0 (0)  0 (0)  0 (0) |

**Supplementary table S5:** Pattern of Bacterial Infections at different timepoints after CAR T cell therapy

|  | **Day 0 – Day 30** | **Day 31 – Day 100** | **Day 101 – Day 180** | **After Day 180** |
| --- | --- | --- | --- | --- |
| **Primary bacteremia without localized organ** | 10 | 1 | 0 | 0 |
| **Localized infection without bacteremia** | 15 | 10 | 5 | 16 |
| **Localized infection with bacteremia** | 0 | 2 | 1 | 0 |

**Supplementary table S6:** Identified viral pathogen at different timepoints after CAR T cell therapy

|  | **Day 0 – Day 30** | **Day 31 – Day 100** | **Day 101 – Day 180** | **After Day 180** |
| --- | --- | --- | --- | --- |
| **Respiratory virus**   - Rhinovirus - Parainfluenza virus - Coronavirus (not include SARS-CoV-2) - Metapneuomovirus - Adenovirus - Influenza virus - Miscellaneous | 5  2  1  1  1  0  0  0 | 8  2  0  1  2  1  1  1 | 8  3  0  1  1  0  1  2 | 6  3  1  0  0  1  0  1 |
| **Norovirus gastroenteritis** | 1 | 0 | 0 | 0 |
| **Cytomegalovirus**   - Viremia - End-organ Disease | 2  2  0 | 1  1  0 | 0  0  0 | 0  0  0 |
| **Polyoma BK virus** | 2 | 1 | 0 | 1 |
| **Herpes zoster virus** | 0 | 0 | 0 | 3 |

*SARS-CoV-2 denotes Severe Acute Respiratory Syndrome coronavirus 2

**Supplementary Table S7:** Univariable analysis for factors associated with bacterial and viral infection after CAR T cell therapy

|  | **Hazard Ratio**  **(95% confidence interval)** | ***P*-Value** |
| --- | --- | --- |
| **Bacterial Infection**   - Age (>60 vs. <60 years) - CAR T product (Tisagenlecleucel vs. Axicabtagene ciloleucel) - Performance Status (>2 vs. 0-1) - History of transplant prior to CAR T cell therapy (Yes vs. No) - History of infection before CAR T cell therapy (Yes vs. No) - Baseline lactate dehydrogenase (Normal vs. High) - Baseline Immunoglobulin G (<400 vs. >400 mg/dL) - Cytokine release syndromes (grade >3 vs. grade 0-2) - Immune Effector Cell Neurotoxicities (grade >2 vs. grade 0-1) - Systemic corticosteroid during CAR T cell (Yes vs. No) | 0.60 (0.29-1.27)  0.68 (0.29-1.59)  1.92 (0.84-4.38)  0.66 (0.27-1.63)  1.12 (0.53-2.36)  0.85 (0.41-1.74)  1.39 (0.63-3.05)  0.84 (0.25-2.83)  1.58 (0.70-0.27)  1.65 (0.75-3.60) | 0.20  0.40  0.12  0.40  0.80  0.70  0.40  0.78  0.27  0.21 |
| **Viral Infection**   - Age (>60 vs. <60 years) - CAR T product (Tisagenlecleucel vs. Axicabtagene ciloleucel) - Performance Status (>2 vs. 0-1) - History of transplant prior to CAR T cell therapy (Yes vs. No) - History of infection before CAR T cell therapy (Yes vs. No) - Baseline lactate dehydrogenase (Normal vs. High) - Baseline Immunoglobulin G (<400 vs. >400 mg/dL) - Cytokine release syndromes (Yes vs. No) - Cytokine release syndromes (grade >3 vs. grade 0-2) - Immune Effector Cell Neurotoxicities (Yes vs. No) - Immune Effector Cell Neurotoxicities (grade >2 vs. grade 0-1) - Systemic corticosteroid during CAR T cell (Yes vs. No) | 1.13 (0.48-2.65)  1.13 (0.47-2.72)  0.65 (0.22-1.92)  0.64 (0.24-1.71)  1.39 (0.63-3.09)  0.77 (0.35-1.70)  5.73 (2.29-14.30)  0.87 (0.33-2.28)  0.30 (0.04-2.26)  1.46 (0.63-3.37)  1.06 (0.43-2.61)  1.39 (0.60-3.22) | 0.80  0.80  0.40  0.40  0.40  0.50  <0.001  0.78  0.24  0.37  0.90  0.44 |

CAR: Chimeric Antigen Receptor

**Supplementary Figure S1:** Serial trend of Procalcitonin Level A) Actual level B) Logarithm Scale (log_10_)


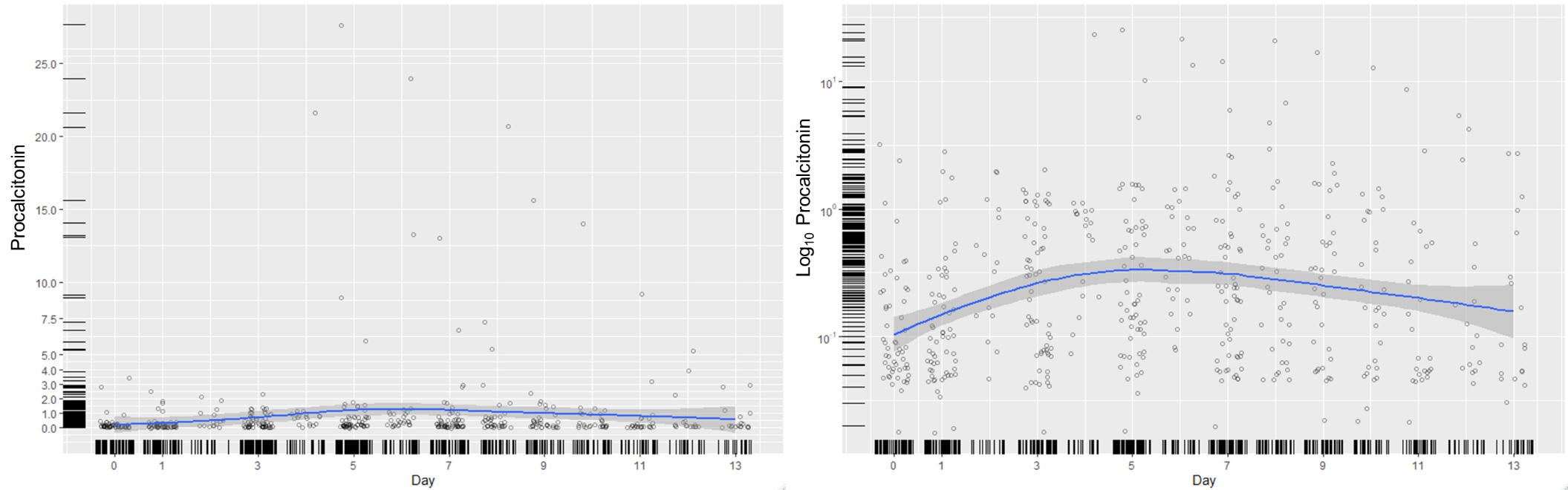


**Supplementary Figure S2:** Serial trend of interleukin-6 Level A) Actual level B) Logarithm Scale (log_10_)


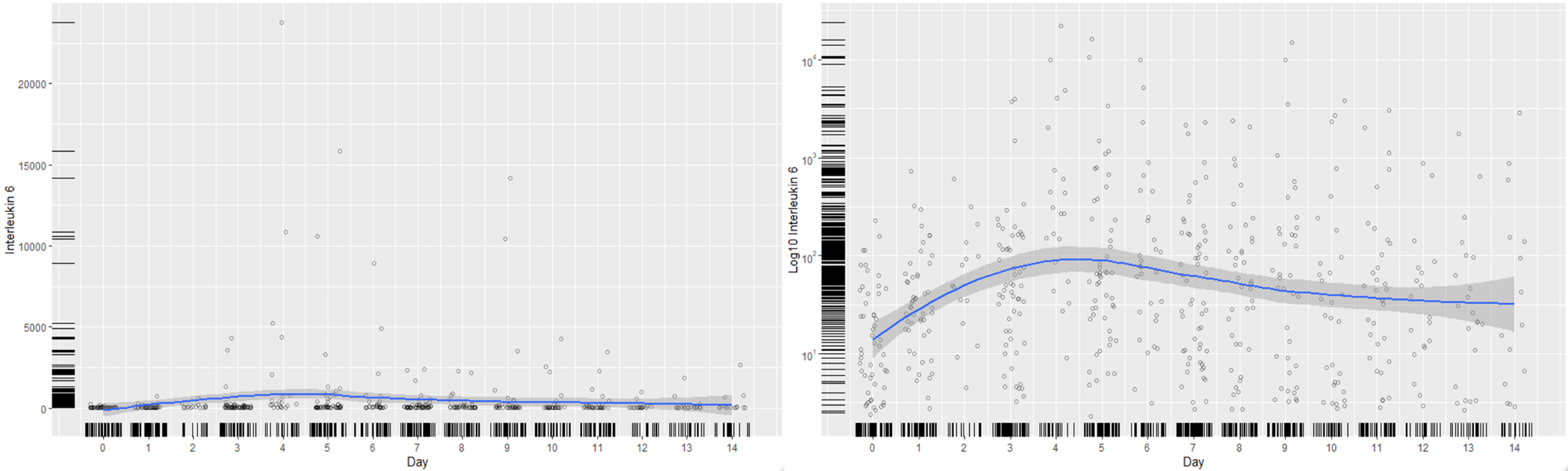

Supplement: Supplementary file 1 — Supplementary Tables [file 41408_2020_346_MOESM1_ESM.docx]
